# Removal of Organic Matter from Tunisian Industrial Phosphoric Acid by Adsorption onto Purified Natural Illite/Kaolinite Clay: Kinetics, Isothermal and Thermodynamic Studies

**DOI:** 10.3390/ma16186228

**Published:** 2023-09-15

**Authors:** Sina Oueriemi, Hedi Ben Amor, Walid Hassen, Bilel Hadrich, Chemseddine Maatki, Karim Kriaa, Lioua Kolsi

**Affiliations:** 1 Laboratory of Processes, Energetic, Environment and Electric Systems, National School of Engineers of Gabes, University of Gabes, Gabes 6072, Tunisia; soueriemi@gmail.com (S.O.); benamorhedi@gmail.com (H.B.A.); 2 Directorate General of Technological Studies, Higher Institute of Technological Studies of Gabes-Tunisia, Gabes 6011, Tunisia; 3 Laboratory of Metrology and Energy systems LR18ES21, National School of Engineering of Monastir, University of Monastir, Ibn Eljazzar Street, Monastir 5019, Tunisia; hassen.walid@gmail.com; 4 Department of Chemical Engineering, College of Engineering, Imam Mohammad Ibn Saud Islamic University (IMSIU), Riyadh 11432, Saudi Arabia; kskriaa@imamu.edu.sa; 5 Department of Mechanical and Industrial Engineering, College of Engineering, Imam Mohammad Ibn Saud Islamic University (IMSIU), Riyadh 11432, Saudi Arabia; casmaatki@imamu.edu.sa; 6 Department of Mechanical Engineering, College of Engineering, University of Ha’il, Ha’il City 81451, Saudi Arabia

**Keywords:** Douiret clay, wet phosphoric acid, organic matter, adsorption, green adsorbent, isotherms, kinetics, thermodynamic

## Abstract

This work aims to use a green, economical and efficient adsorbent to remove organic matter from Tunisian industrial wet phosphoric acid (WPA: 52% P_2_O_5_). For this purpose, a natural and abundant clay is extracted from the Douiret, Tataouine deposit in southern Tunisia. This clay is being tested for the first time as an adsorbent in WPA medium. The raw clay and purified clay are analysed using standard analytical techniques such as Fourier transform infrared spectroscopy, X-ray diffraction, and BET methods. The results show that the raw clay is a mixture of illite and kaolinite, with other mineral impurities, mainly quartz. Organic matter adsorption tests show that the purified clay exhibits greater effectiveness than raw clay. The parametric study with purified clay indicates that temperature, contact time, and clay dosage strongly influence organic matter adsorption. The highest adsorption occurs at 60 °C after 50 min, reaching 56% with 8 g of purified clay per kg of WPA. Among several recognised models, the pseudo-second-order kinetic model and the Sips isotherm model are the most suitable for modelling the experimental data. This study suggests that Douiret clay can be considered an effective, inexpensive and environmentally friendly adsorbent for eliminating organic matter in industrial phosphoric acid.

## 1. Introduction

Phosphoric acid ranks as the second-most extensively manufactured acid on a global scale [1]. According to the IFA “International Fertilizer Industry Organization”, the world production of phosphoric acid was estimated at 59,936 t of P_2_O_5_, in 2021. In addition to its primary role in the fertiliser sector, phosphoric acid also has various other uses, such as in food processing, detergents, medicine, and water treatment.

Phosphoric acid is industrially manufactured from phosphate rock in two main ways: thermal or wet processes [2]. The thermal process enables the production of high-quality phosphoric acid, but at a very significant energy cost and with severe environmental impacts [3]. Therefore, manufacturers prefer the wet process, which accounts for approximately 90% of the global production of phosphoric acid [4]. During this process, the phosphate rock reacts with a powerful mineral acid such as hydrochloric, nitric, or sulfuric acid [5]. Among these, sulfuric acid is predominantly utilised due to its lower cost, simple operation, and ability to exploit lower-quality phosphate rocks [6]. Unfortunately, undesirable impurities from phosphate rock such as fluoride, heavy metals, and organic matter (OM) are inevitably leached out with the phosphorus [7]. Some of these elements affect the acid quality and limit its end use. Indeed, regulatory standards for industrial phosphoric acid quality are gradually becoming stricter, particularly in applications related to fertilisers, detergents, food processing, pharmaceuticals, and electronics [8].

Organic matter includes humic substances, aromatic compounds, phthalates, and many other carboxylic acids [7]. Based on its geological source, the organic matter content in sedimentary phosphates rocks varies between 1% and 3.5% [9]. This confers a dark colour to wet acid and considerably limits its uses. Furthermore, OM can induce odour issues during fertiliser production [10] and create stable toxic complexes with heavy metals and other elements in phosphoric acid [11]. Some of these undesirable complexes may accumulate in both soil and water before eventually reaching humans through the various stages of the food chain [8,12]. In addition, organic matter can strongly impact the efficiency of solvent extraction due to its interaction with organic solvent [13,14]. Therefore, it is crucial to decrease the concentration of the organic compounds in wet phosphoric acid to achieve the required acid quality for the production of environmentally friendly fertilisers, and various industrial and food-grade phosphate derivatives. Several techniques have been deployed to eliminate organic substances from wet phosphoric acid, such as chemical oxidation [7], ion exchange [15], solvent extraction [16], ionic flotation [17], membrane processes [18], extraction with impregnated resins [19], and crystallisation [20]. Nevertheless, the use of these techniques has been consistently limited because of their high energy requirements, significant costs linked to organic solvents, oxidants, and resins, as well as the inherent risk of secondary pollution [21]. Conversely, adsorption is often preferred in many cases thanks to its considerable effectiveness, broad applicability, and simple implementation [22].

Activated carbon is widely acknowledged to be the most extensively employed adsorbent due to its exceptional adsorption properties [23]. However, its practical use is limited by its high cost, lack of environmental friendliness, and heavy dependence on imports, particularly in countries like Tunisia. Consequently, research has focused on finding new green and sustainable alternatives, such as generating activated carbon from agricultural residue [11,24] or using natural clays. Clay materials are among the abundant and inexpensive natural resources that can be used and easily modified thanks to their height exchange cationic capacity [25]. Smectite clay, in particular, has been the subject of some papers [26]. In this study, we conducted the first-ever test on an illite/kaolinite clay extracted from the Douiret, Tataouine deposit in the southern region of Tunisia, commonly used in cosmetic and ceramic applications [27,28]. Our objective is to evaluate its effectiveness as an adsorbent for organic matter found in Tunisian wet phosphoric acid. After characterising raw (RD) and purified clays (PD), as well as industrial phosphoric acid, a series of experiments were performed in batch mode. The experiments involved varying the applied adsorbent dosage, adjusting the adsorption duration, and controlling the temperature. The primary objective was to attain the highest level of adsorption performance. Equilibrium adsorption data were modelled using the Freundlich, Redlich–Peterson and Sips isotherm models. Additionally, the pseudo-first-order, pseudo-second-order and intraparticular diffusion kinetic models were used to identify the kinetic parameters and to describe the adsorption process. Thermodynamic functions were also calculated in order to shed light on the nature of the sorption process.

## 2. Materials and Methods

### 2.1. Materials

**Figure 1:**
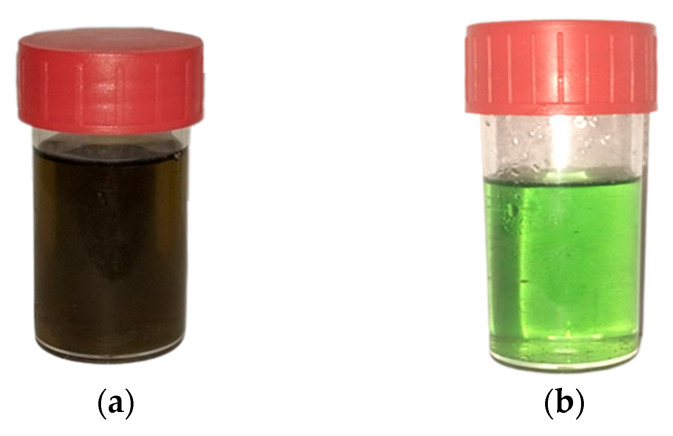
Photos of concentrated wet phosphoric acid sample: (**a**) before treatment and (**b**) after treatment with purified Douiret clay.

This study incorporates three distinct groups of material, specifically:

Concentrated wet phosphoric acid manufactured by the Tunisian Chemical Group situated in Gabes, south of Tunisia. The blackish green colouration (Figure 1) of this acid (containing 52% P_2_O_5_) is attributed to its elevated concentration of organic matter.

The raw clay selected was mined from the Douiret, Tataouine deposit, which is one of the major clay reserves in Tunisia [29].

Analytical-grade chemical reagents are utilised without further purification, and distilled water is employed to prepare different solutions. Sodium hydroxide (NaOH, purity > 99%), sulfuric acid (H_2_SO_4_ 98% wt) and potassium bromide (KBr, purity ≥ 99%) were sourced from Merck. Potassium dichromate (K_2_Cr_2_O_7_, purity > 99%) was purchased from Labokem. Mohr’s salt ((NH_4_)_2_Fe(SO_4_)_2_.6H_2_O, purity = 99%) and sodium citrate (HOC(COONa)(CH_2_COONa)_2_ 2H_2_O, purity = 99%) were supplied by Sigma-Aldrich. Bromocresol green and phenolphthalein, coloured indicators used to measure P_2_O_5,_ were obtained from Atlanticlabo.

### 2.2. Methods

#### 2.2.1. Clay Purification

The refining process for untreated raw clay involves successive stages of grinding, dispersing, sedimentation, and centrifugal separation. Initially, the untreated raw clay is smashed using an agate mortar and then sifted with stainless-steel sieves to collect particles smaller than 160 µm. These particles are spread out in distilled water with a mass ratio of 10 wt%. [30]. Next, the mixture is subjected to intense mechanical stirring for a minimum duration of 4 h to ensure effective homogenisation. Following this, the mixture is moved to a 2 L graduated cylinder for sedimentation at room temperature. The time required to recover the finest fraction (with a particle size ≤ 2 µm) is determined according to Stokes’ law [31], expressed as:

(1) $$t=190\frac{x}{d^{2}}$$

where t is the sedimentation time (min), x is the siphoned depth (cm), and d is the particle diameter (µm).

Supernatants containing clay-sized particles are pipetted and then centrifuged at 7000 rpm. The resulting purified clay is then dried at a temperature of 60 °C for a duration of 24 h, followed by additional grinding and sieving through a 125 µm sieve. The clay obtained through this process is used throughout this work.

#### 2.2.2. Industrial Phosphoric Acid Characterisation

The main characteristics of WPA are determined as follows:

The P_2_O_5_ content is determined via an alkalimetric assay using a standard sodium hydroxide solution with bromocresol green and phenolphthalein as a colour indicator [20].

The elemental composition is determined using an atomic absorption spectrophotometer coupled with inductively coupled mass spectrometry [32] at the Tunisian Chemical Group laboratories in Gabes.

The analysis of fluorine is carried out using the potentiometric method with a specific fluoride ion electrode (Thermo Scientific Orion, Dual StarpH/ISE, Waltham, MA, USA) in the presence of a buffer based on sodium citrate [33].

The OM content is quantified using a redox titrimetric method involving a heated mixture of K_2_Cr_2_O_7_ in a sulfuric acid solution. The excess of Cr_2_O_7_^2−^ is back titrated with a standardised solution of Fe^2+^ [20].

#### 2.2.3. Clay Characterisation

The elemental analyses of raw clay are performed by X-ray fluorescence (XRF) using an Oasis 9900 spectrometer (Thermo Fisher, Waltham, MA, USA). Ignition loss is measured by calcination at 1000 °C.

The mineralogical analysis is performed using a Bruker AXS D8 X-ray diffractometer (XRD) (Billerica, MA, USA) operating on monochromatic Kα1 radiation from copper (40 kV, 40 mA, λ = 0.15406 nm). The Fourier-transform infrared (FTIR) spectra of raw clay and purified clay are recorded using a Bruker Alpha ATR (Billerica, MA, USA) spectrometer operating in the interval of 400–4000 cm^−1^ at a resolution of 2 cm^−1^. Potassium bromide (KBr) is used as a substance to dilute the samples. The Quantachrome instrument from Nova Instruments, Boynton Beach, FL, USA (a division of Anton Paar Quanta Tec Inc., Boynton Beach, FL, USA) is employed to generate nitrogen adsorption–desorption isotherms in order to determine the specific surface area. The data are recorded and evaluated with Quantachrome NovaWin version 11.06 software. Before conducting the analysis, all samples are subjected to vacuum degassing process at 105 °C for 12 h.

#### 2.2.4. Adsorption Experiments

Organic matter adsorption experiments are conducted in batch mode within a specified temperature range of 25 to 75 °C. Different doses of PD are added to 100 g of WPA, and the mixtures are magnetically stirred at a speed of 400 rpm. This procedure follows the steps described by Hamza and EL-Naggar in their published work [11,34]. Afterward, the samples are collected at the desired time and subjected to centrifugation at a speed of 6500 rpm for a duration of 3 min. Subsequently, the supernatants are analysed to determine the residual concentration of organic matter. This analysis enables the removal efficiency (R_E_ in %) and OM adsorbed amount (q_t_ in mg·g^−1^) to be computed. These calculations are performed using Equations (2) and (3).

(2) $$R_{E}=\frac{\left(C_{O}-C_{t}\right)}{C_{O}}\cdot 100$$

(3) $$q_{t}=\frac{\left(C_{O}-C_{t}\right)}{m}\cdot w$$

Here, C_0_ and C_t_ (mg·kg^−1^) represent the organic matter concentrations of phosphoric acid initially and at time t, respectively, m (g) is the mass of the clay, and w (kg) is the mass of the phosphoric acid sample. Once equilibrium is achieved, C_t_ and q_t_ transform into C_e_ and q_e_, respectively. Additionally, it is important to mention that, for the sake of accuracy, all experiments are conducted in triplicate.

#### 2.2.5. Kinetics Model

Kinetic studies can determine the contact time and expression of the rate law required to achieve optimal adsorbent efficiency. This allows the implementation of the adsorption process on a large scale. To characterise the adsorption kinetics of organic matter on purified clay, the pseudo-first-order and pseudo-second-order kinetic models and the intraparticular diffusion model are adopted to fit the experimental data conducted at various temperatures.

- Pseudo-first-order kinetic model: Its linear equation was defined by Lagergren as follows [35]:

(4) $$\ln\left(q_{e}-q_{t}\right)=-k_{1}\cdot t+\ln\left(q_{e}\right)$$

- Pseudo-second-order Kinetic Model: Its linear equation can be expressed as follows [35]:

(5) $$\frac{t}{q_{t}}=\frac{t}{q_{e}}+\frac{1}{k_{2}\cdot q_{e}^{2}}$$

- Intraparticular Diffusion Model: This model, suggested by Weber, is used to examine the mechanism of the adsorption process. Its linear equation is as follows [35]:

(6) $$q_{t}=k_{in}\cdot t^{0.5}+C$$

Here, q_e_ and q_t_ (mg·g^−1^) are the quantities of organic matter adsorbed at equilibrium and at time t. k_1_ (min^−1^) and k_2_ (g·mg^−1^·min^−1^) represent the rate constants of the pseudo-first-order and pseudo-second-order reactions, respectively. k_in_ (mg·g^−1^·min^−0.5^) is the intraparticular diffusion rate constant and C is the intercept that reflects the significance of the external diffusion.

#### 2.2.6. Adsorption Isotherm Models

The amount of adsorbate fixed on the adsorbent surface at equilibrium (q_e_) is connected to the remaining concentration of the same adsorbate in the liquid phase at equilibrium (C_e_) at a constant temperature, as described by the adsorption isotherm. According to Hinz (2001), the plot of the distribution coefficient (K_D_ = q_e_/C_e_) against q_e_ forms a linear line. The direction of the slope on this line assists in the identification of the isotherm shape and the selection of an appropriate model from those available in literature.

In the present study, three nonlinear models, namely, the Freundlich, the Redlich–Peterson, and the Sips models, are applied to fit the experimental isotherm data.

- Freundlich Model: This empirical approach is frequently employed to describe the adsorption on heterogeneous systems possessing active sites with various affinities. Its expression is given by Equation (7) [36].

(7) $$q_{e}=K_{F}\cdot C_{e}^{1/n}$$

Here, K_F_ (mg^l−(l-n)^·L^l/n^/g) is the Freundlich constant, and 1/n is the heterogeneity factor.

- Redlich–Peterson Model: This hybrid model combines the Langmuir and Freundlich isotherms, and may be adopted for either homogeneous or heterogeneous systems. It is expressed as [36]:

(8) $$q_{e}=\frac{K_{R}\cdot C_{e}}{1+a_{R}\cdot C_{e}^{g}}$$

where K_R_ (L·g^−1^) and a_R_ (L·mg^−1^)^g^ are parameters of the Redlich–Peterson isotherm and g is a dimensionless constant.

- Sips Model: This isotherm model, similar to the Redlich–Peterson model, is a combination of the Freundlich and Langmuir models and is applied for heterogeneous systems. The sips model equation is given by [36,37]:

(9) $$q_{e}=\frac{q_{\max}{\left(b\cdot C_{e}\right)}^{1/n}}{1+{\left(b\cdot C_{e}\right)}^{1/n}}$$

The maximum adsorption potential is represented by q_max_ (mg·g^−1^), and the Sips isotherm constant is denoted by b (L·mg^−1^), while the Sips model exponent is indicated by n.

Thanks to advancements in computer technology, the nonlinear regression approach has been gaining significant popularity in recent years. In this approach, the model parameters are obtained by minimising the squared error between the experimental values and the predictions made by the model.

The correlation coefficient (R^2^), Chi-square (χ^2^) and the sum of squared errors (SSE) are among the techniques used to select appropriate models. All these parameters are calculated using the following equations [38]:

(10) $$R^{2}=1-\frac{{\sum \left(q_{e,\mathrm{mod}}-q_{e,\exp}\right)}^{2}}{{\sum \left(q_{e,\exp}-q_{e,mean}\right)}^{2}}$$

(11) $$\chi^{2}=\sum \frac{{\left(q_{e,\mathrm{mod}}-q_{e,\exp}\right)}^{2}}{q_{e,\mathrm{mod}}}$$

(12) $$SSR={\sum \left(q_{e,\mathrm{mod}}-q_{e,\exp}\right)}^{2}$$

Here, q_e,exp_ is the experimental value of adsorption capacity, q_e_,_mod_ is the value predicted by the tested model using solver 2, while q_e,mean_ (mg·g^−1^) is the mean of the experimental values.

#### 2.2.7. Adsorption Thermodynamics

- Thermochemical Parameters

To check the feasibility of the adsorption process, a thermodynamic study is necessary. The impact of temperature on organic matter adsorption allows the identification of thermodynamic variables such as enthalpy (ΔH°), entropy (ΔS°), Gibbs free energy (ΔG°) and the dimensionless distribution coefficient K_D_ [11,39]. The variables are quantified using the following equations:

(13) $$K_{D}=\frac{q_{e}}{C_{e}}$$

(14) $$\Delta G^{{}^{\circ}}=-R\cdot T\cdot \ln\left(K_{D}\right)$$

(15) $$Ln\left(K_{D}\right)=\frac{\Delta S^{{}^{\circ}}}{R}-\frac{\Delta H^{{}^{\circ}}}{R\cdot T}$$

R represents the ideal gas constant with a value of 8.314 J·mol^−1^·K^−1^, where T is the adsorption temperature (in K). The determination of ΔS° and ΔH° can be accomplished using Van ’t Hoff’s plot, as outlined in Equation (15).

- Activation Energy

According to Arrhenius’ law, the rate constant (k) is linked to temperature by Equation (16) [40]:

(16) $$k=A_{0}\cdot e^{\left(-\frac{E_{a}}{R\cdot T}\right)}$$

In our case, k (g·mg^−1^·min^−1^) is equivalent to k_2_, which stands for the pseudo-second-order constant, A_0_ represents the temperature-independent rate constant (g·mg^−1^·min^−1^), and E_a_ (J·mol^−1^) is the activation energy of adsorption.

The linear form of Arrhenius’ law is:

(17) $$ln\left(k_{2}\right)=-\frac{E_{a}}{R\cdot T}+\ln\left(A_{0}\right)$$

E_a_ and A_0_ are defined, respectively, on the basis of the slope and the y-intercept of the plot showing ln(k_2_) as a function of 1/T.

## 3. Results and Discussion

### 3.1. Industrial Phosphoric Acid Characterisation

The analysis of WPA shows a percentage of P_2_O_5_ content of 51.63 wt% and an organic matter content of 570 ppm (Table 1). The other main impurities are aluminium, magnesium, iron, and fluorine. The most detected heavy metals are chromium and zinc. It should be mentioned that the organic composition content and the percentage of P_2_O_5_ content were determined before each experimental campaign.

### 3.2. Douiret Clay Characterisation

**Figure 2:**
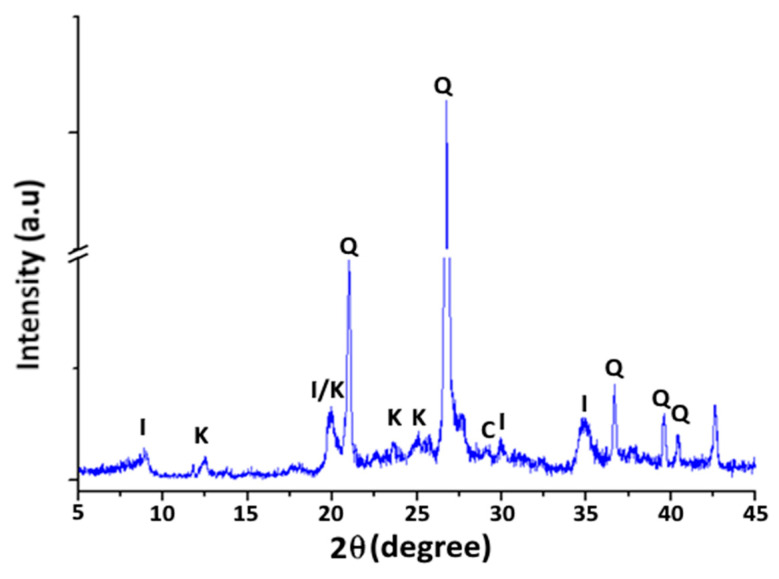
X-ray diffraction pattern of raw clay sample.

- Chemical Analysis of Raw Clay: Table 2 presents the results of chemical analysis of the raw clay sample using X-ray fluorescence. As intended, the main constituents of RD are silica (SiO_2_) and alumina (Al_2_O_3_). It can be observed that the iron content is quite high, which is characteristic of Tunisian clays [41]. In contrast, the calcium content is very low, indicating a limited presence of calcite content. This is further validated through a negative hydrochloric acid test.

- Mineralogical Analysis: The X-ray diffraction patterns of raw clay in Figure 2 show that the raw material is mostly made up of illite (I), as identified by reflections at 9.8 Å (2Ɵ = 8.94°); 4.47 Å (2Ɵ = 19.95°); 2.98 Å (2Ɵ = 29.31°) and 2.56 Å (2Ɵ = 34.91°) and kaolinite (K) as indicated by the appearance of reflections at 7.1 Å (2Ɵ = 12.47°); 3.76 Å (2Ɵ = 23.59°) and 3.55 Å (2Ɵ = 25.06°) [41]. The spectrum highlights the presence of a non-swelling phase (impurities) primarily consisting of quartz, characterised by reflections 4.27 Å (2Ɵ = 20.76°); 3.33 Å (2Ɵ = 26.72°); 2.44 Å (2Ɵ = 36.69°) and 2.27 Å (2Ɵ = 39.53°). Finally, a peak reflection at 3.04 Å (2Ɵ = 29.31°) indicates the presence of calcite in trace amounts [42].

**Figure 3:**
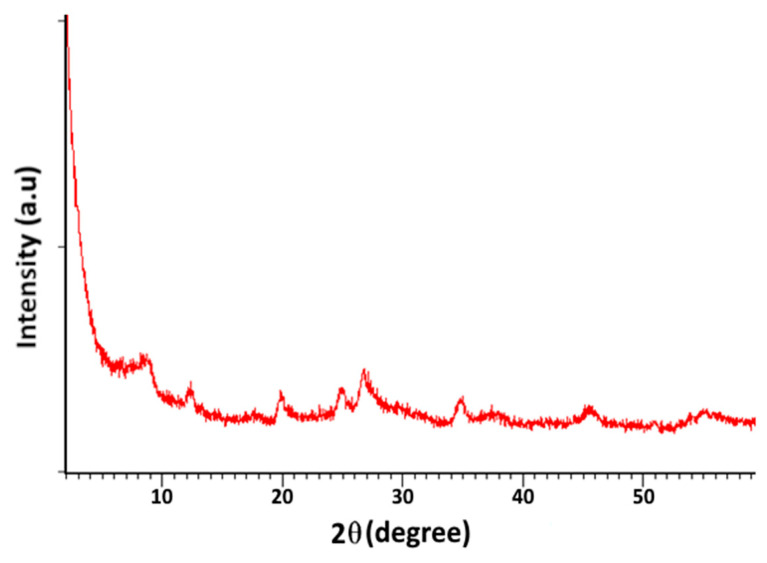
X-ray diffraction pattern of purified clay sample.

The X-ray diffractogram analysis of the purified sample (Figure 3) reveals an intensification of illite’s characteristic peaks (10.022 and 2.58°A). Furthermore, a more significant decrease in the level of the characteristic quartz peaks is observed, indicating a reduction in the concentrations of associated minerals in the samples after the purification process.

**Figure 4:**
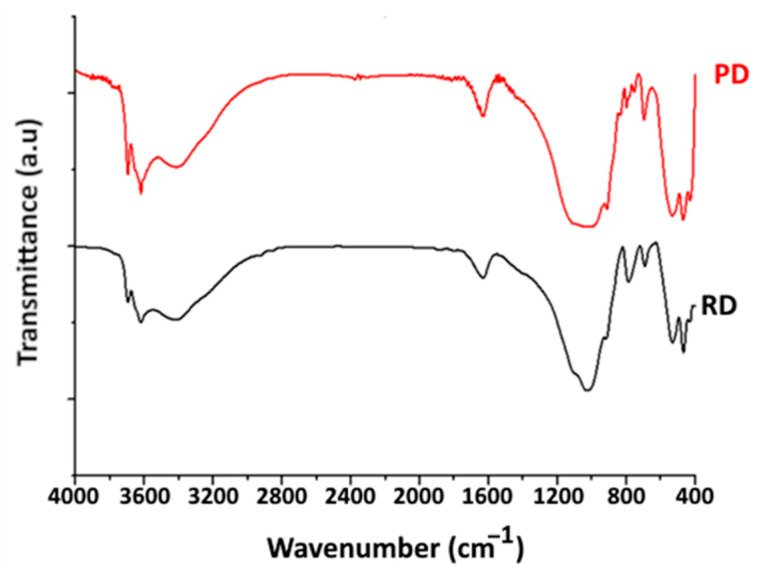
Infrared spectra of raw and purified Douiret clay samples.

- Functional Group Analysis: The existence of a band situated within the 3200–3800 cm^−1^ range is recorded in the infrared spectrum of the raw material, as depicted in (Figure 4). Peaks at 3621 and 3419 cm^−1^ are related to the stretching vibrations of the OH groups of the octahedral layer. The first peak corresponds to AlMgOH and/or Al_2_OH [28]. The second peak is linked to the deformation vibrations of water molecules. The peak centred near 1632 cm^−1^ is linked to the deformation vibrations of the H_2_O molecules adsorbed between the sheets. Furthermore, Al-OH bending bands in the 690–705 cm^−1^ range are a distinctive feature of kaolinite and other di-octahedral clays [28]. The identification of illite clay using infrared techniques has been reported to be quite difficult [43]. Quartz, the most important non-clay mineral, is found at 798 cm^−1^ [28]. The wide band of 1032 cm^−1^ is related to the Si-O stretching vibration. The bending bands for Si-O-Al are located at 540–555 cm^−1^, and for Si-O-Si, they are at 425–480 cm^−1^.

By comparing the infrared spectra of two clay samples (RD) and (PD), similar absorption bands are detected. However, a significant decrease in the intensity of the peak associated with quartz (798 cm^−1^) is observable. Conversely, there is a noticeable increase in the signals corresponding to the kaolinite mineral (700 cm^−1^), with the spectral range indicating bending vibrations of Si-O-Al bonds (532 to 470 cm^−1^) and elongation vibrations attributed to the hydroxyl (OH) groups within the octahedral layer (3700 to 3620 cm^−1^). These results validate the effectiveness of the clay purification.

**Figure 5:**
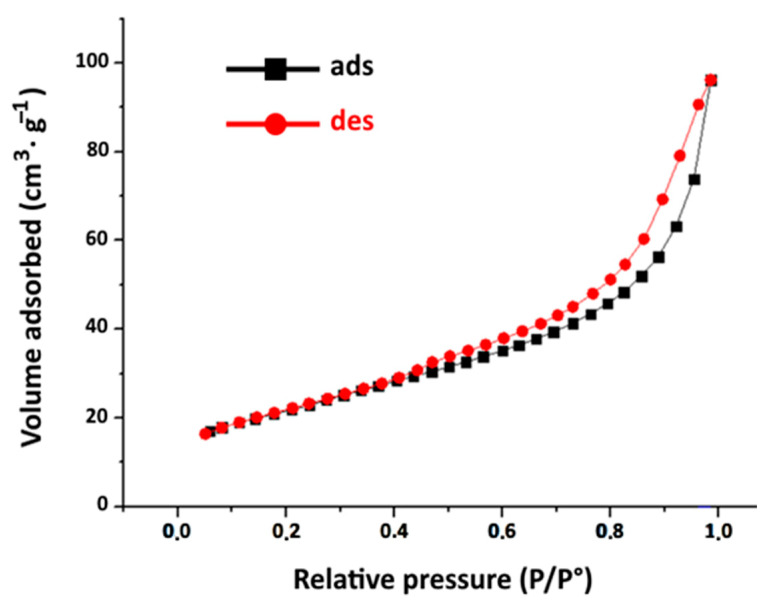
Nitrogen adsorption–desorption isotherm at 77 K of Raw Clay.

- BET Specific Surface Area and Porosity Analysis: Figure 5 displays the nitrogen adsorption–desorption isotherms in raw clay determined at 77 K. According to the nomenclature of the IUPAC (International Union of Pure and Applied Chemistry), these isotherms can be classified as type IV, exhibiting a hysteresis cycle denoted as H_3_. This designation implies a mesoporous clay structure [44].

The specific surface are, and porosity of the clay are evaluated, respectively, using BET method and the Barrett–Joyner–Halenda model, as illustrated in Table 3. The mean pore size suggests a mesoporous structure of the raw clay.

### 3.3. Adsorption Study

#### 3.3.1. Effect of Purification on Efficiency of Organic Matter Removal

**Figure 6:**
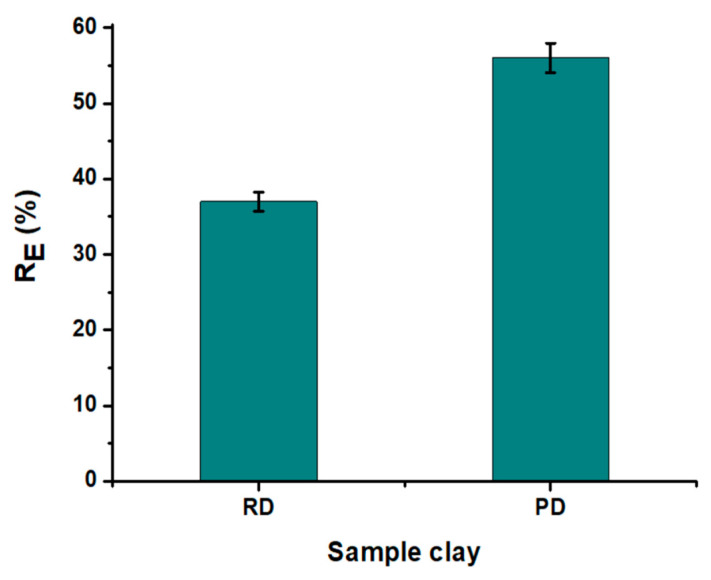
Influence of clay purification on the organic matter removal efficiency (T = 60 °C, time = 60 min, and clays dose = 8 g·kg^−1^).

To compare the organic matter retention efficiency in WPA of both raw and purified clays, adsorption tests are conducted under similar conditions. These conditions include a clay dose of 8 g·kg^−1^, a reaction temperature of 60 °C, and an adsorption time of 60 min with an agitation velocity of 400 rpm. According to Figure 6, organic matter removal is enhanced by purification, resulting in an increase from 38% with raw clay to 56% in purified clay. Therefore, only the purified clay is retained for additional examination.

#### 3.3.2. Effect of Clay Dose and Temperature

**Figure 7:**
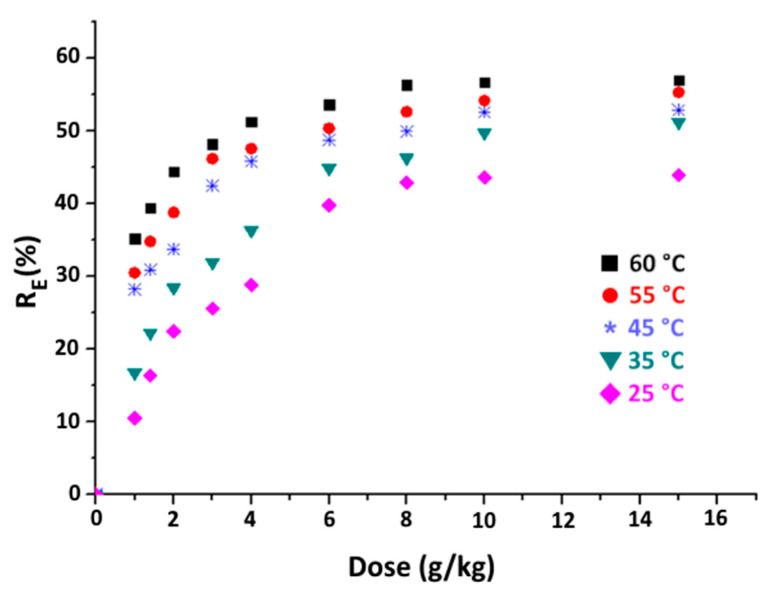
Variation in organic matter removal efficiency versus purified clay dose at different temperatures (time = 60 min).

The variation in organic matter removal efficiency as a function of the purified clay dose (1 to 15 g·kg^−1^) at various temperatures is shown in Figure 7. According to this figure, the removal efficiency of organic matter improves when the adsorbent dose is increased from 1 to 8 g·kg^−1^. This is primarily due to the enhanced availability of active sites when increasing the adsorbent dose. However, once the adsorbent dose goes beyond 8 g·kg^−1^, the increase in the percentage of organic matter elimination becomes less sensitive to further increases in adsorbent dose. This behaviour can be attributed to the creation of clusters by the adsorbent particles leading to a reduction in both the surface area and the number of active sites on the adsorbent material [45].

Furthermore, higher temperatures enhance the elimination of organic matter. Consequently, with a purified clay dose of 8 g·kg^−1^, the percentage of OM elimination increases from 42% to 57% with an increase in temperature from 25 to 60 °C. This shift can be explained by the fact that organic matter adsorption on purified clay below 60 °C is an endothermic process. Additionally, the mobility of organic species in the acid medium is improved by the decrease in viscosity with increasing temperature, which in turn facilitates their transfer to the adsorbent.

**Figure 8:**
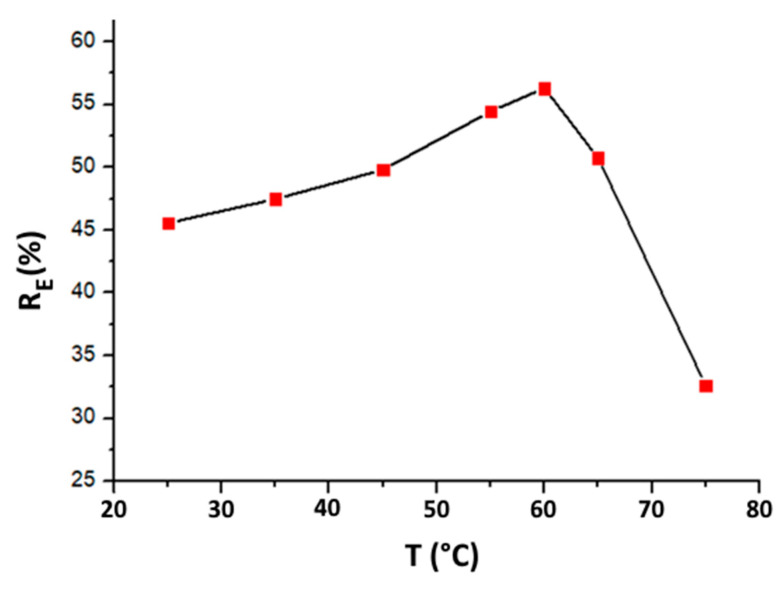
Temperature effect on removal efficiency of organic matter (time = 60 min, purified clay dose = 8 g·kg^−1^).

Nevertheless, as depicted in Figure 8, the efficiency of MO adsorption declines at temperatures above 60 °C. In fact, increasing temperature (beyond 60 °C) promotes the desorption phenomenon, and hence reduces the efficiency of adsorption. Based on the obtained results, 60 °C is identified as the best temperature to achieve optimal organic matter adsorption onto purified clay.

### 3.4. Effect of Contact Time

**Figure 9:**
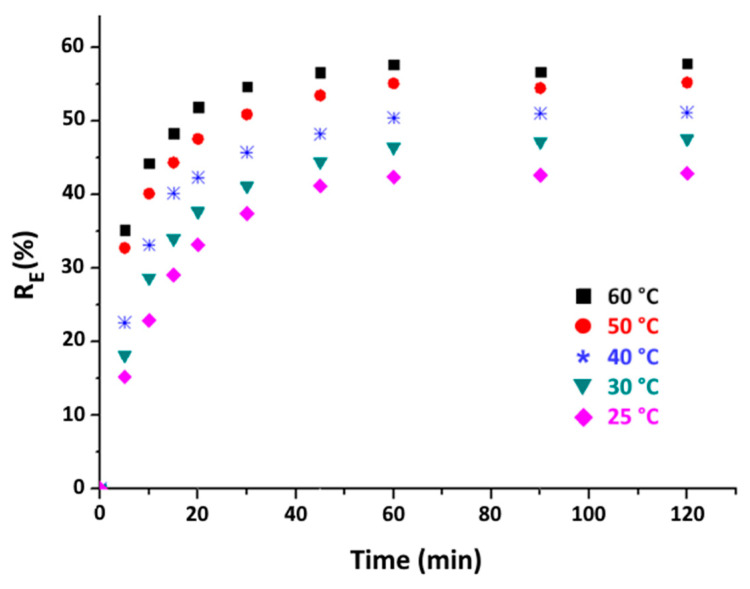
Effect of contact time on organic matter removal efficiency at different temperatures (purified clay dose = 8 g·kg^−1^).

It is well known that contact time is a key parameter in all transfer phenomena, especially in the adsorption field. Therefore, the effect of contact time on the efficiency of eliminating organic matter is investigated at various temperatures (25 to 60 °C), while using the same optimal dosage of 8 g·kg^−1^ of purified clay. The results, as depicted in Figure 9, illustrate that, initially, the adsorption process occurs rapidly at each specified temperature. This phenomenon is attributed to the abundance of active sites available on the clay surface. However, as the adsorption process progresses, the number of available active sites decreases, leading to a slower adsorption rate. A state of equilibrium is achieved at around the 50 min. Furthermore, Figure 9 indicates that, regardless of the contact time, better organic matter removal performance is achieved at 60 °C. This observation aligns with the findings highlighted in the previous section.

### 3.5. Kinetic Results

**Figure 10:**
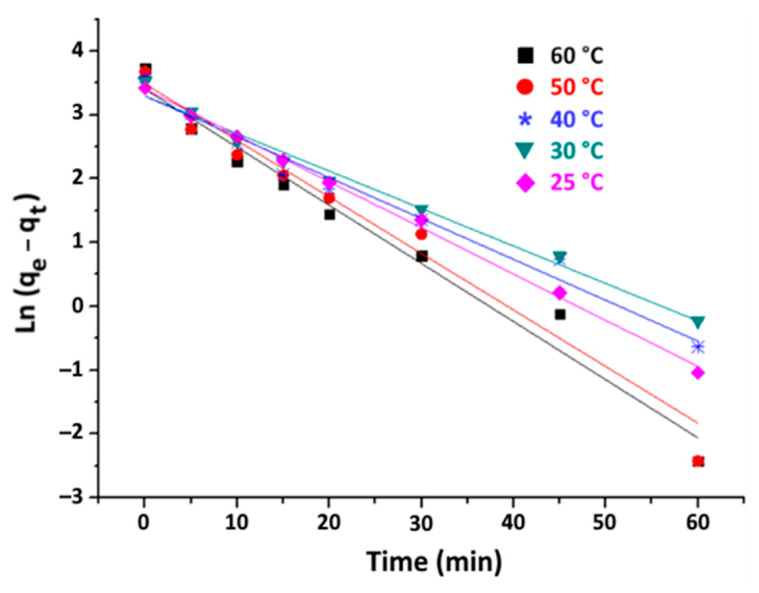
Pseudo-first-order plots of organic matter adsorption on purified clay at different temperature (purified clay dose = 8 g·kg^−1^).

**Figure 11:**
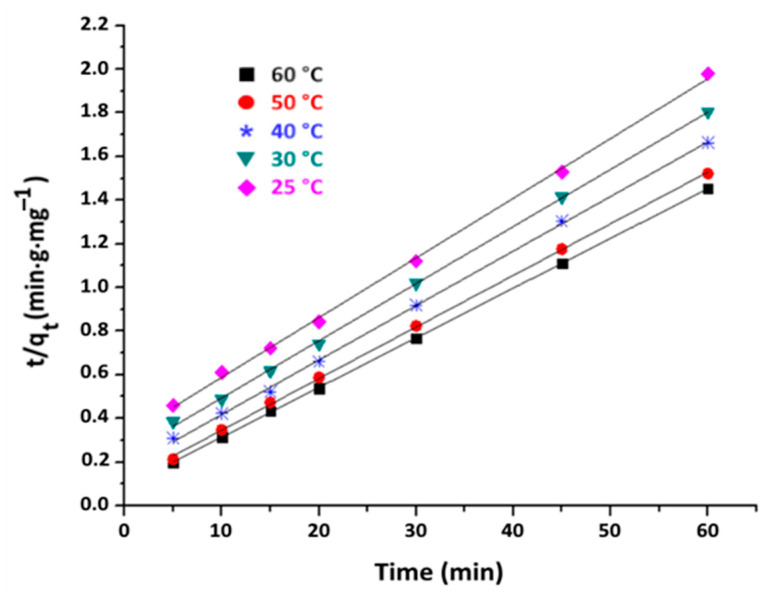
Pseudo-second-order plots of organic matter adsorption on purified clay at different temperature (purified clay dose = 8 g·kg^−1^).

The linear plots of ln(q_e_ − q_t_) versus time and t/q_t_ versus time are shown in Figure 10 and Figure 11, respectively.

The parameters of each model are calculated based on the slopes and y-intercepts of the obtained straight-line curves. These values are presented in Table 4 along with related R^2^ correlation coefficients.

From the data in Table 4, the R^2^ values associated with the pseudo-second-order model are close to 1 for all tested temperatures. Moreover, the q_e_ values predicted by this model are in closer agreement with the experimental values compared to those predicted by the first-order model. Consequently, it can be inferred that the kinetics of adsorption of organic matter on purified clay are governed by a pseudo-second-order model. This kind of behaviour is frequently observed when clays interact with organic substances [46].

According to Weber and Morris [47,48], the adsorption of a fluid solute on the surface of the adsorbent occurs in four successive steps:

- Migration of the adsorbate from the bulk liquid phase to the boundary layer of the liquid film, which is bound to the solid particle.
- Transfer of the adsorbate through the liquid layer to the external surface of the adsorbent (external diffusion).
- Diffusion of the adsorbate inside the adsorbent pores (intraparicular diffusion).
- Finally, adsorption of the solute on the active site.

The first and fourth steps tend to proceed faster compared to the second and third steps. Consequently, these stages are not considered in the overall kinetics of the adsorption process [49].

**Figure 12:**
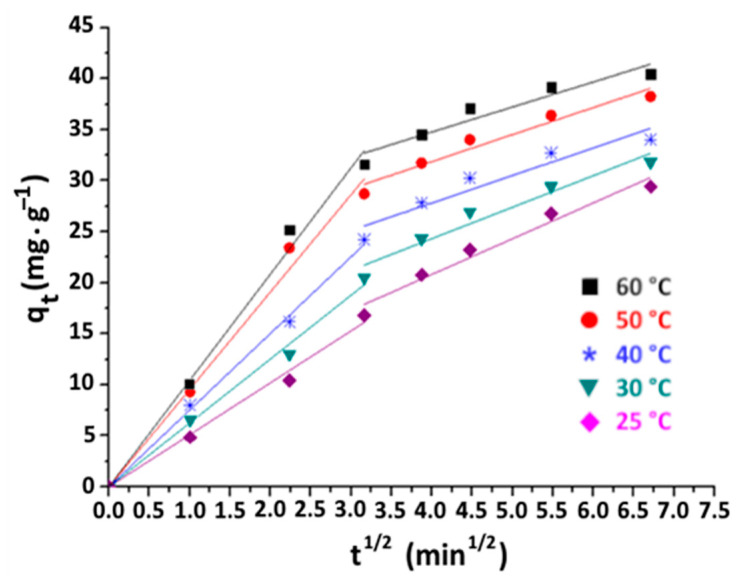
Diffusion plots of organic matter adsorption on purified clay at different temperatures (purified clay dose = 8 g·kg^−1^).

In the case of the adsorption of organic matter onto purified clay, the plots of q_t_ versus t^1/2^ (Figure 12) show two linear trends. These trends indicate that the adsorption procedure takes place in two distinct phases. The initial phase occurs within the first 10 min, and is associated with the external diffusion process. The second phase occurs between 10 and 50 min, corresponding to the process of intraparticle diffusion within the clay particles.

The parameters of each step are deduced based on the slopes and y-intercepts of the obtained straight-line curves. Their numerical values are tabulated in Table 5, with their corresponding R^2^ correlation coefficients.

It can be seen from Table 5 that the increase in temperature slightly affects the kinetics of intraparticular diffusion, with ki_nt2_ decreasing from 4.26 to 3.26 mg·g^−1^·min^−1/2^. In contrast a strong increase in the values of k_int1_ and C with temperature can be noted. Indeed, k_int1_ rises from 5.09 to 10.32 mg·g^−1^·min^−1/2^ and C increases from 3.81 to 21.76 mg·g^−1^ when varying the temperature from 25 to 60 °C. This finding suggests that the temperature elevation promotes external diffusion due to the reduced viscosity of the medium. The results indicate a dual control mechanism for the adsorption process involving both intraparticle and external diffusion.

### 3.6. Adsorption Isotherm Models

**Figure 13:**
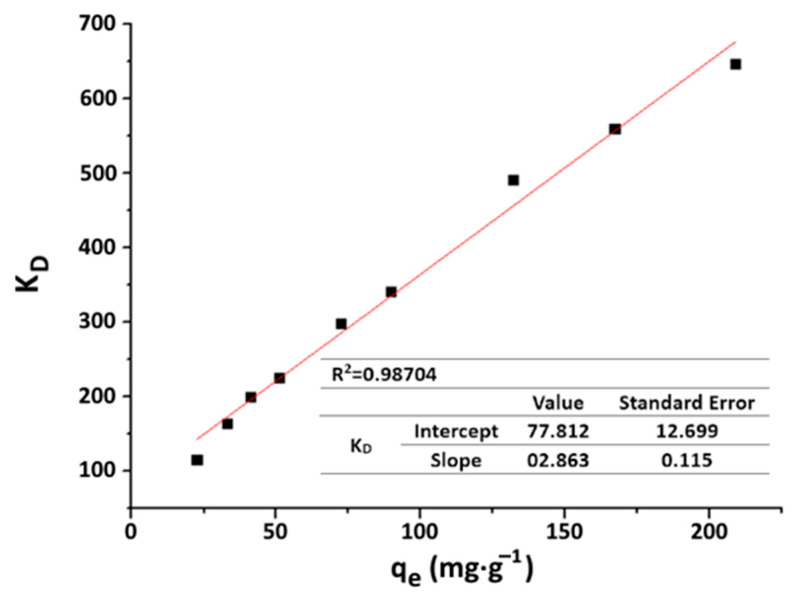
Plot of distribution coefficient vs. adsorption capacity.

The plot of the distribution coefficient (K_D_) against the adsorption capacity (q_e_) in Figure 13 shows a linear curve with a positive slope. This linear curve signifies that the isotherm falls under the “S”-type classification. The presence of this S-shaped curve can be ascribed to the collaborative adsorption of organic molecules or to the competitive interactions among the various impurities present in WPA. Based on this result, the Freundlich, Redlich–Peterson and Sips models are adopted to fit the experimental data obtained at equilibrium. The Langmuir model, a commonly used adsorption model, is excluded, because it is unsuitable for S-type isotherms involving multiple types of activated sites.

**Figure 14:**
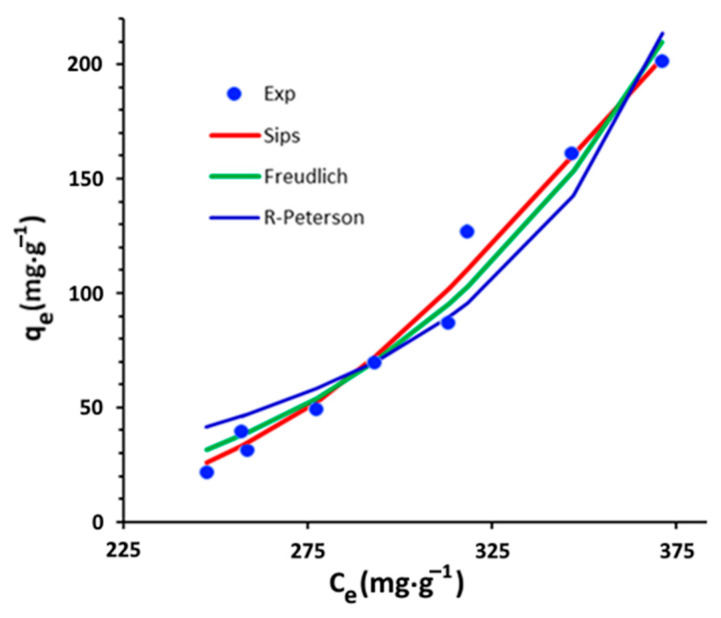
Equilibrium isotherm of organic matter absorbed onto purified clay at 60 °C.

The experimental isotherm at 60 °C and the nonlinear plots of the Freundlich, Redlich–Peterson and Sips isotherms models for the adsorption of organic matter by purified clay are illustrated in Figure 14. It can be observed that the Sips and Freundlich models appear to be more appropriate than the Redlich–Peterson model for describing the adsorption isotherm. This observation is validated by the error values presented in Table 6. The Sips model yielded a higher R^2^ value, as well as lower values of χ^2^ and SSR error, indicating a maximum adsorption capacity of 364.5 mg·g^−1^.

### 3.7. Thermodynamic Parameters and Activated Energy of Adsorption

**Figure 15:**
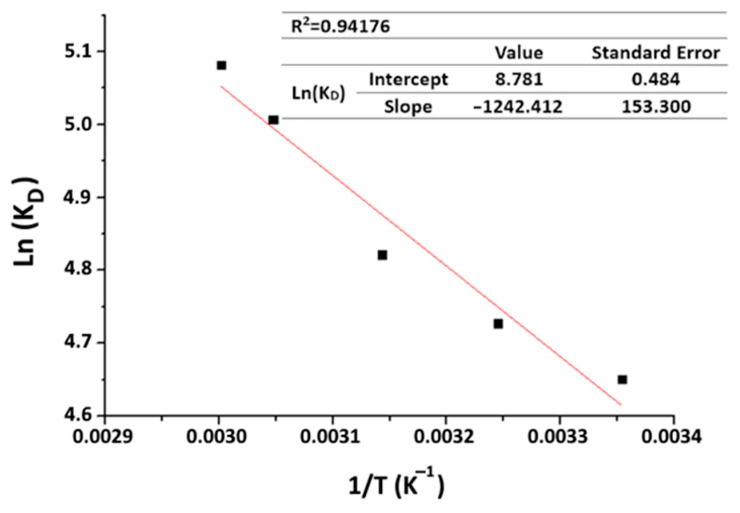
Van ’t Hoff plot for organic matter adsorption on purified clays (purified clay dose = 8 g·kg^−1^; time = 60 min).

The Van ’t Hoff plot (Figure 15) allows the calculation of ΔH° and ΔS° from the slope and y-intercept of the obtained straight-line curve. The estimated values are listed in Table 7.

Negative ΔG° values can be observed, and their magnitude decreases with increasing temperature. This confirms the spontaneous character and the beneficial effect of temperature on MO adsorption. The positive ΔH° values imply an endothermic adsorption process, which aligns with the experimental results. The value, which is lower than 40 KJ·mol^−1^, denotes the physical nature of the adsorption. The positive ΔS° points to increasing disorder at the solid–liquid interface during adsorption.

**Figure 16:**
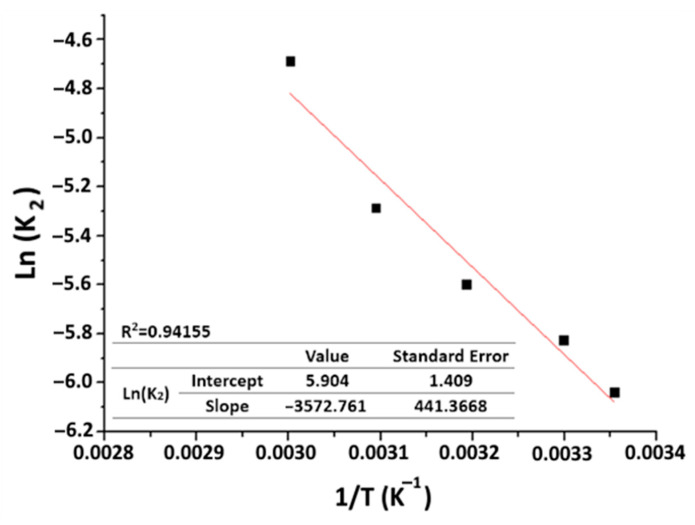
Arrhenius plot for organic matter adsorption on purified clays (purified clay dose = 8 g·kg^−1^; time = 60 min).

Based on the graph depicted in Figure 16 (ln(k_2_) versus (1/T)), the activation energy (E_a_) is estimated to be 29,704 kJ·mol^−1^, while the constant (A_0_) is found to be 366.720 g·mg^−1^·min^−1^. Equation (18) can be used to express the correlation between the pseudo-second-order constant (k_2_) and temperature.

(18) $$k_{2}=366.720\;\exp\left(-\frac{29704.259}{R\cdot T}\right)=366.720\;\exp\left(-\frac{3572.8}{T}\right)$$

Understanding of the nature of adsorption is provided by the level of activation energy. Weak activation energy values, ranging between 5 and 50 kJ·mol^−1^, indicate physical adsorption, while higher activation energy values, ranging between 60 and 800 kJ·mol^−1^, indicate chemical adsorption [50,51]. The E_a_ value obtained in this study confirms the physical nature of the organic matter adsorption by purified clay.

## 4. Comparison of Douiret Clay with Bentonite Clays

Table 8 compares the maximum adsorption capacity of organic matter by purified Douirat clays and various bentonite clays (currently used in WPA purification) [26,34,52]. It can be seen that Douiret’s illite/kaolinite exhibits significantly higher efficiency. Therefore, the Douiret clay proves to be a highly promising alternative to bentonite clay for industrial phosphoric acid purification.

## 5. Conclusions

In this study, the adsorption of organic matter from Tunisian industrial wet phosphoric acid (51.63 wt% P_2_O_5_) was investigated using a local illite/kaolinite clay extracted from the Douiret, Tataouine region. The results indicated that the purification of the raw clay improved the reduction of organic matter content, which increased from 38% to 56%. A parametric study revealed that the optimal removal of organic matter occurred at a temperature of 60 °C, using a dose of purified clay of 8 g·kg^−1^, and a contact time of 50 min. The kinetic study indicated that the organic matter adsorption followed a pseudo-second-order kinetic model and was simultaneously controlled by the intraparticular and external diffusion mechanisms.

The experimental data at equilibrium at 60 °C were accurately described by both the Sips and Freundlich models, with a maximum adsorption capacity of 364.47 mg·g^−1^ being estimated by Sips model.

This value significantly surpassed those obtained for bentonite-type clays. The thermodynamic study revealed that the organic matter adsorption was an endothermic, physical, and spontaneous process. Finally, the Douiret clay seemed to be more efficient at removing organic matter from wet phosphoric acid compared to bentonite clays. Douiret clay offers great potential as a sustainable, environmentally friendly, inexpensive, and efficient material for the purification of wet phosphoric acid.

## Figures and Tables

**Table 1:** Chemical composition of industrial wet phosphoric acid.

Element	OM	Al	Fe	Mg	F	Cd	Cr	Mn	V	Zn
Content (ppm)	570	4127	3420	7522	3700	24	312	41	82	157

**Table 2:** Chemical analysis of raw clay.

Constituent	SiO_2_	Al_2_O_3_	Fe_2_O_3_	CaO	SO_3_	K_2_O	Na_2_O	Cl	MgO	P_2_O_5_	LOI	Total
Content (wt%)	53.27	19.29	8.81	traces	0.79	4.06	0.12	0.07	2.18	0.12	10.86	99.57

**Table 3:** Specific surface area and porous properties of the raw Douiret clay.

Parameter	BET Surface Area (m^2^·g^−1^)	Mean Pore Diameter (A°)	Total Pore Volume (cm^3^·g^−1^)
Value	76.784	31.336	0.127

**Table 4:** Parameters of the pseudo-first-order and pseudo-second-order kinetic models for organic matter adsorption on purified clay (purified clay dose = 8 g·kg^−1^).

T (°C)	q_exp_ (mg·g^−1^)	Pseudo First Order			Pseudo Second Order		
		R^2^	q_e_ (mg·g^−1^)	k_1_ (min^−1^)	R^2^	q_e_ (mg·g^−1^)	k_2_ (g·mg^−1^·min^−1^)
60	41.36	0.961	26.28	0.0806	0.999	43.86	0.0092
50	39.50	0.963	26.08	0.0712	0.999	42.37	0.0051
40	36.58	0.948	26.19	0.0615	0.999	40	0.0037
30	34.02	0.983	27.27	0.0644	0.999	38.16	0.0029
25	30.66	0.998	29.04	0.0697	0.998	36.90	0.0024

**Table 5:** Parameters of the intraparticular diffusion model for organic matter adsorption onto purified clay at different temperatures (purified clay dose = 8 g·g^−1^).

T (°C)	First Step		Second Step		
	k_int 1_ (mg·g^−1^·min^−1/2^)	R^2^	k_int 2_ (mg·g^−1^·min^−1/2^)	C (mg·g^−1^)	R^2^
60	10.32	0.9946	3.26	21.76	0.9673
50	9.31	0.9964	3.31	18.65	0.9788
40	7.51	0.9980	3.63	13.33	0.9642
30	6.26	0.9930	3.83	9.04	0.9642
25	5.09	0.9903	4.26	3.81	0.9862

**Table 6:** Characteristic parameters of different isotherm models.

Model	Equation	Parameter	Unit	Value
Freundlich	(7)	K_F_	(mg·g^−1^)^1−1/n^	2.289 × 10^−10^
		n	-	0.214
		R^2^	-	0.969
		χ^2^	-	12.413
		SSR	-	973.907
Redlich–Peterson	(8)	g	-	−11.451 × 10^−3^
		K_R_	mg·g^−1^	−11.067 ×10^−4^
		a_R_	kg·mg^−1^	−1.072
		R^2^	-	0.929
		χ^2^	-	30.611
		SSR	-	2247.477
Sips	(9)	n	-	0.145
		q_max_	kg·mg	364.473
		b	(kg·mg)^g^	2.784 × 10^−3^
		R^2^	-	0.982
		χ^2^	-	7.338
		SSR	-	570.452

**Table 7:** Thermodynamic parameters of organic matter adsorption on purified clay.

T (K)	ΔG° (kJ·mol^−1^)	ΔH° (kJ·mol^−1^)	ΔS° (J·mol^−1^·K^−1^)
298.15	−11.527	10.329	73.008
308.15	−12.111		
318.15	−12.752		
328.15	−13.660		
333.15	−14.075		

**Table 8:** Comparison of Douiret clay to some bentonite clays.

Adsorbent	Temperature (°C)	Sorbent Dosage	Contact Time (min)	q_max_ (mg·g^−1^)	Isotherm Model	Reference
Tunisian bentonite (Gafsa)	50 30	-	30	18.52 14.71	Langmuir	[26]
Tunisian bentonite (EL-Hamma)	35	20 g·L^−1^	90	17.26	Langmuir	[34]
Algerian bentonite	30	8 g·L^−1^	95	153.28	Langmuir	[52]
Tunisian illite (Douiret)	60	8 g·kg^−1^	50	364.47	Sips	This study

## Data Availability

Not applicable.

## References

[1] Grand View Research (2023). Phosphoric Acid Market Size, Grand View Research—Share & Trends Analysis Report.

[2] Gilmour R. (2019). Phosphoric acids and phosphates. Kirk-Othmer Encyclopedia of Chemical Technology.

[3] Kouzbour S., Gourich B., Gros F., Vial C., Allam F., Stiriba Y. (2019). Comparative analysis of industrial processes for cadmium removal from phosphoric acid: A review. Hydrometallurgy.

[4] Ober J.A. (2018). Mineral Commodity Summaries.

[5] Chen M., Li Z., Huang P., Li X., Qu J., Yuan W., Zhang Q. (2018). Mechanochemical transformation of apatite to phosphoric slow-release fertilizer and soluble phosphate. Process. Saf. Environ. Prot..

[6] Becker P. (1983). Phosphates and Phosphoric Acid: Raw Materials, Technology, and Economics of the Wet Process.

[7] Zhou Y., Zheng G., Long Y., Liu Z., Tao C., Liu R. (2022). Advanced oxidation processes for wet-process phosphoric acid: Enhanced phosphorus recovery and removal of organic matters. Hydrometallurgy.

[8] Taha M.H., Abdel Maksoud S.A., Ali M.M., El Naggar A.M., Morshedy A.S., Elzoghby A.A. (2019). Conversion of biomass residual to acid-modified bio-chars for efficient adsorption of organic pollutants from industrial phosphoric acid: An experimental, kinetic and thermodynamic study. Int. J. Environ. Anal. Chem..

[9] Baturin G.N., Kochenov A.V. (2001). Uranium in phosphorites. Lithol. Miner. Resour..

[10] Theys T. Influence of the rock impurities on the phosphoric acid process, products and some downstream uses. Proceedings of the IFA Technical Committee Meeting.

[11] El Naggar A.M., Ali M.M., Abdel Maksoud S.A., Taha M.H., Morshedy A.S., Elzoghby A.A. (2019). Waste generated bio-char supported co-nanoparticles of nickel and cobalt oxides for efficient adsorption of uranium and organic pollutants from industrial phosphoric acid. J. Radioanal. Nucl. Chem..

[12] Fayiga A.O., Nwoke O.C. (2016). Phosphate rock: Origin, importance, environmental impacts, and future roles. Environ. Rev..

[13] Mellah A., Benachour D. (2007). Adsorption of Heavy Metals from Industrial Phosphoric Acid by Algerian Activated Bentonite. Model. Ann. Chim. Sci. Mat..

[14] Dissanayake C.B., Chandrajith R. (2009). Introduction to Medical Geology.

[15] Koopman C., Witkamp G.J. (2002). Ion exchange extraction during continuous recrystallization of CaSO_4_ in the phosphoric acid production process: Lanthanide extraction efficiency and CaSO_4_ particle shape. Hydrometallurgy.

[16] Awwad N.S., El-Nadi Y.A., Hamed M.M. (2013). Successive processes for purification and extraction of phosphoric acid produced by wet process. Chem. Eng. Process. Process Intensif..

[17] Chang M.Y., Juang R.S. (2004). Adsorption of tannic acid, humic acid, and dyes from water using the composite of chitosan and activated clay. J. Colloid Interface Sci..

[18] Bendada A., Meniai A.H., Bencheikh L.M. (2001). Modeling of Phosphoric Acid Purification by Liquid-Liquid Extraction. Chem. Eng. Technol..

[19] Kabay N., Demircioğlu M., Ekinci H., Yüksel M., Sağlam M., Streat M. (1998). Extraction of Cd (II) and Cu (II) from phosphoric acid solutions by solvent-impregnated resins (SIR) containing cyanex 302. React. Funct. Polym..

[20] Hannachi A., Habaili D., Chtara C., Ratel A. (2007). Purification of wet process phosphoric acid by solvent extraction with TBP and MIBK mixtures. Sep. Purif. Technol..

[21] Olyaie E., Banejad H., Afkhami A., Rahmani A., Khodaveisi J. (2012). Development of a cost-effective technique to remove the arsenic contamination from aqueous solutions by calcium peroxide nanoparticles. Sep. Purif. Technol..

[22] Taha M.H., El-Maadawy M.M., Hussein A.E.M., Youssef W.M. (2018). Uranium sorption from commercial phosphoric acid using kaolinite and metakaolinite. J. Radioanal. Nucl. Chem..

[23] Monser L., Amor M.B., Ksibi M. (1999). Purification of wet phosphoric acid using modified activated carbon. Chem. Eng. Process. Process Intensif..

[24] Abderrahim N., Djellabi R., Amor H.B., Fellah I., Giordana A., Cerrato G., Bianchi C.L. (2022). Sustainable purification of phosphoric acid contaminated with Cr (VI) by Ag/Ag_3_PO_4_ coated activated carbon/montmorillonite under UV and solar light: Materials design and photocatalytic mechanism. J. Environ. Chem. Eng..

[25] Parmar M., Thakur L.S. (2013). Heavy Metal Cu, Ni, and Zn: Toxicity, Health Hazards and Their Removal Techniques by Low-Cost Adsorbents: A Short Overview. Int. J. Plant Anim. Environ. Sci..

[26] Khoualdia B., Loungou M., Elaloui E. (2017). Adsorption of Organic Matter from Industrial Phosphoric Acid (H3PO4) onto Activated Bentonite. Arab. J. Chem..

[27] Bouaziz S. (2005). Les Matières Premières Naturelles du Gouvernorat de Tataouine: Caractérisation et Utilisations.

[28] Mahmoudi S., Bennour A., Meguebli A., Srasra E., Zargouni F. (2016). Characterization and traditional ceramic application of clays from the Douiret region in South Tunisia. Appl. Clay Sci..

[29] Ouaja M., Barale G., Philippe M., Ferry S. (2011). Occurrence of an in situ fern grove in the Aptian Douiret Formation, Tataouine area, South-Tunisia. Geobios.

[30] Brown G. (1982). Crystal Structures of Clay Minerals and Their X-ray Identification.

[31] Gueu S., Finqueneisel G., Zimny T., Bartier D., Yao B.K. (2019). Physicochemical characterization of three natural clays used as adsorbent for the humic acid removal from aqueous solution. Adsorp. Sci. Technol..

[32] Aboulhassane A., El Idrissi A.N., Bounou Y., Zakaria D. (2019). 29% P_2_O_5_ phosphoric acid desulphation: Enhancing concentration unit performance. Am. J. Analyt. Chem..

[33] Gouider M., Feki M., Sayadi S. (2009). Separative recovery with lime of phosphate and fluoride from an acidic effluent containing H3PO4, HF and/or H2SiF6. J. Hazard. Mater..

[34] Hamza W., Chtara C., Benzina M. (2013). Retention of organic matter contained in industrial phosphoric acid solution by raw Tunisian clays: Kinetic equilibrium study. J. Chem..

[35] Husien S., El-Taweel R.M., Mohamed N., Abdel-Aziz A., Alrefaey K., Elshabrawey S.O., Mostafa N.G., Said L.A., Fahim I.S., Radwan A.G. (2023). Potentials of algae-based activated carbon for the treatment of M. orange in wastewater. Case Stud. Chem. Environ. Eng..

[36] Abin-Bazaine A., Trujillo A.C., Olmos-Marquez M. (2022). Enlightenment of the Adsorption Phenomenon: Adsorption Isotherms in Wastewater Treatment. Wastewater Treatment.

[37] Trinh V.T., Nguyen T.M.P., Van H.T., Hoang L.P., Nguyen T.V., Ha L.T., Vu X.H., Pham T.T., Quang N.V., Nguyen X.C. (2020). Phosphate adsorption by silver nanoparticles-loaded activated carbon derived from tea residue. Sci. Rep..

[38] Almeida-Naranjo C.E., Aldás M.B., Cabrera G., Guerrero V.H. (2021). Caffeine elimination from synthetic wastewater using magnetic fruit peel composites: Investigating material properties, isotherm behavior, and kinetic patterns. Environ. Chall..

[39] Mubarak M.F., Mohamed A.M.G., Keshawy M., Abd elMoghny T., Shehata N. (2022). Adsorption of heavy metals and hardness ions from groundwater onto modified zeolite: Batch and column studies. Alex. Eng. J..

[40] Winayu B.N.R., Mao W.H., Chu H. (2022). Combination of rGO/S, N/TiO_2_ for enhancing visible light-driven toluene photocatalytic degradation. Sustain. Environ. Res..

[41] Jarraya I., Fourmentin S., Benzina M., Bouaziz S. (2010). VOC Adsorption on Raw and Modified Clay Materials. Chem. Geol..

[42] Amri I., Ouakouak A., Hamdi W., Srasra E., Hamdi N. (2022). Removal of Non-Steroidal Drug from Waste Water Using Synthetic Zeolites from Illito-Kaolinitic Clays. Water Air Soil Pollut..

[43] Ritz M., Vaculikova L., Plevova E. (2011). Application of Infrared Spectroscopy and Chemometric Methods to Identification of Selected Minerals. J. Anal. Chem..

[44] Mendhe V.A., Mishra S., Khangar R.G., Kamble A.D., Kumar D., Varma A.K., Singh H., Kumar S., Bannerjee M. (2017). Organo-petrographic and pore facets of Permian shale beds of Jharia Basin with implications for shale gas reservoir. J. Earth Sci..

[45] El-Zahhar A.A., Ali M.M., Ahmed A.M., Khalifa M.E., Abdel-Bary E.M. (2015). Removal of Iron from Wet-Process Phosphoric Acid Using Titanium Silicate-Polymer Composite. CTAIJ.

[46] Hamza W., Chtara C., Benzina M. (2016). Purification of 54% industrial phosphoric acid through Fe-pillared bentonite. Environ. Sci. Pollut. Res..

[47] Weber W.J., Morris J.C. (1963). Kinetics of Adsorption on Carbon from Solution. J. Sanit. Eng. Div..

[48] Bizi M., El Bachra F.E. (2021). Transport of carbamazepine, ciprofloxacin, and sulfamethoxazole in activated carbon: Solubility and correlations between molecular structure and diffusional characteristics. Molecules.

[49] Guo Z., Liu X., Huang H. (2015). Kinetics and Thermodynamics of Reserpine Adsorption onto Strong Acidic Cationic Exchange Fiber. PLoS ONE.

[50] Nollet H., Roels M., Lutgen P., Van der Meeren P., Verstraete W. (2003). Removal of PCBs from wastewater using fly ash. Chemosphere.

[51] Hameed B.H., Ahmad A.A., Aziz N. (2007). Isotherms, kinetics and thermodynamics of acid dye adsorption on activated palm ash. J. Chem. Eng..

[52] Boualia A., Mellah A., Aissaoui T.T., Menacer K., Silem A. (1993). Adsorption of Organic Matter Contained in Industrial H_3_PO_4_ onto Bentonite: Batch-Contact Time and Kinetic Study. Appl. Clay Sci..

